# Deciphering Planktonic Bacterial Community Assembly in the Storage Reservoir of the Long-Distance Water Diversion Project

**DOI:** 10.3390/microorganisms13020465

**Published:** 2025-02-19

**Authors:** Yingying Yang, Liguo Chen, Nianxin Wan, Ailing Xu, Ning Ding, Zhiwen Song

**Affiliations:** 1 School of Environmental and Municipal Engineering, Qingdao University of Technology, Qingdao 266520, China; yangyingyingqd@163.com (Y.Y.);; 2 Shandong Water Transfer Project Operation and Maintenance Center, Jinan 250199, China; 3 Jihongtan Reservoir Management Station of Shandong Water Transfer Project Operation and Maintenance Center, Qingdao 266111, China

**Keywords:** co-occurrence network patterns, environmental driving factors, planktonic bacterial community assembly, seasonal dynamics, storage reservoir

## Abstract

Storage reservoirs are crucial components of long-distance water diversion projects, where water diversion may lead to changes in microbial diversity and community structure. Seasonal variations also drive alterations in microbial communities. However, the way that microbes assemble under the combined effects of water diversion and seasonal variations in the storage reservoir has not been extensively studied. Jihongtan Reservoir is the terminal storage reservoir of the Yellow River to Qingdao Water Diversion Project (YQWD), which had an average annual water diversion period exceeding 290 days in recent years. In this study, 16S rDNA amplicon sequencing was used to investigate the seasonal dynamics and assembly of planktonic bacterial communities during the water diversion period in Jihongtan Reservoir. The results indicate that planktonic bacteria were able to maintain stable diversity across all four seasons, while the community structure underwent significant seasonal succession. Water temperature (WT) was found to be the primary driving environmental factor influencing the seasonal dynamic of planktonic bacterial communities. Co-occurrence network patterns of planktonic bacterial communities varied across different seasons, particularly in spring and winter. The spring network displayed the most complexity, showcasing the highest connectivity and greater stability. In contrast, the winter network was simpler, exhibiting lower local connectivity but higher global connectivity and lower stability. The analysis of the neutral community model and null model revealed that the relative importance of deterministic and stochastic processes in governing planktonic bacterial community assembly varies seasonally. Stochastic processes (dispersal limitation) are more prominent in spring, summer, and autumn, while deterministic processes (heterogeneous selection) play a greater role in winter. This study is essential for gaining a comprehensive understanding of the effects of water diversion projects and offers valuable references for the assessment of other similar projects.

## 1. Introduction

The unpaired spatial and temporal distribution of water resources leads to regional water scarcity, and inter-regional water transfer is commonly adopted globally to alleviate this contradiction in water usage [1,2,3]. The YQWD is a large-scale, inter-basin, and long-distance water transfer initiative designed to meet urban water supply needs, agricultural water requirements, and ecological water replenishment. Jihongtan Reservoir is the terminal storage reservoir of the YQWD and is the largest water source for Qingdao City, China. During peak periods, it supplies up to 90% of the total urban water supply for the city. As a storage reservoir, Jihongtan Reservoir impedes the hydro-morphological and ecological continuum in the water conveyance canal [4]. These changes can selectively influence microbial growth and metabolism, result in the restructuring of microbial communities, and affect related biogeochemical cycles [5,6]. For instance, the reduced flow velocity leads to the sedimentation of particulate matter, which improves the light conditions and enhances primary productivity, resulting in the reconstruction of complex food web structures [7].

Water diversion projects can cause significant disturbances to ecosystems in both the donor and recipient areas, as well as along the diversion routes [8,9,10,11], leading to complex, delayed, and extensive ecological effects [12]. The receiving area, as an important component of the water diversion project, has been studied for changes before and after water diversion. For instance, researchers have studied the phytoplankton in the regulating reservoirs of water diversion projects before and after water diversion and found changes in local biodiversity, community structure, and stability as a result of water diversion [13,14,15]. Meanwhile, recent reports indicate that seasonal variation significantly influenced the diversity, taxonomic composition, and structure of microbial communities in water transfer projects and even found that the effects of water diversion in a certain period are not as substantial as those of environmental factors [16]. The YQWD was completed and put into operation in 1989. Initially, it had an annual average of 71 days of water transfer, while the average annual water diversion period has exceeded 290 days since 2015. It is evident that the water diversion period for Jihongtan Reservoir now significantly surpasses the non-diversion period. Therefore, it is essential to conduct an in-depth study of the microorganisms in Jihongtan Reservoir during the water diversion period.

Planktonic bacteria are a vital component of aquatic ecosystems, exhibiting high genetic diversity and significantly contributing to the global biogeochemical cycles of carbon, nitrogen, and other elements [17]. Understanding diversity patterns and microbial community assembly mechanisms is a primary topic in microbial ecology [18]. It is generally accepted that community assembly is governed by both deterministic processes (niche-related processes) and stochastic processes (neutral-related processes) [19,20]. Based on niche theory, deterministic processes assert that microbial community composition is shaped by both abiotic factors (such as temperature, pH, and nutrients) and biotic factors (such as competition and predation) [21]. In contrast, the neutral theory emphasizes that stochastic processes are the main determinants of the microbial community, including random speciation, death, dispersal, and ecological drift [22]. Studying the bacterial co-occurrence patterns facilitates our understanding of the ecological processes affecting the microbial community. Co-occurrence networks are ecological networks in which species are represented as nodes and their relationships as links [23]. The topological characteristics of the network can help infer the interactions between species (such as competition, predation, mutualism, etc.) and can be applied to investigate the complexity, connectivity, and stability of microbial communities [24]. Additionally, co-occurrence networks can be used to identify keystone taxa [25]. These species are highly connected groups and play an essential role in the stability of microbial communities [26].

This study utilizes 16S rDNA amplicon sequencing technology to investigate the dynamics of planktonic bacterial communities and their ecological regulation mechanisms in Jihongtan Reservoir during the water diversion period. Our study primarily focuses on the following aspects: (1) to profile the seasonal succession of planktonic bacterial community structure in the reservoir; (2) to uncover the relationship between environmental factors and planktonic bacterial communities and identify the key environmental drivers of planktonic bacterial seasonal succession; (3) to elucidate the co-occurrence patterns of planktonic bacterial communities across different seasons and identify keystone species; and (4) to determine the assembly of planktonic bacterial communities across four seasons.

## 2. Materials and Methods

### 2.1. Study Site, Sample Collection, and Environmental Variables

Samples were collected in Jihongtan Reservoir (36°20′55′′~36°23′31′′ N, 120°12′27′′~120°14′55′′ E), a drinking water reservoir in Qingdao City, Shandong Province, China. The reservoir is the largest man-made dammed plain reservoir in Asia, with a volume of approx. 1.57 × 10^8^ m^3^, a design water level of 14.2 m, a length of 14.2 km, and a surface area of 14.1 km^2^. Jihongtan Reservoir serves as the terminal storage reservoir of YQWD, a large-scale inter-basin water transfer project with a total length of 290 km and a designed annual water diversion capacity of 1.03 × 10^8^ m^3^. Upon its completion, Jihongtan Reservoir predominantly diverted allogenic water from the Yellow River. As a result of enhanced water resource scheduling strategies, Jihongtan Reservoir now benefits from a diversified array of water supply sources. Supplementing the Yellow River inflow, the reservoir receives water from the Yangtze River, a component of the Eastern Route of the South-to-North Water Diversion Project (ESNWD), and surplus water from Xiashan Reservoir (XR) during the rainy season. These water resources are transported to Jihongtan Reservoir via the main conduit of the YQWD. The multi-water resource replenishment to Jihongtan Reservoir every month during the experimental period is presented in Table S1. Jihongtan Reservoir is the primary drinking water source for Qingdao City, supplying over 90% of the water utilized by the urban population. It has a designed daily average water supply capacity of 0.71 × 10^6^ m^3^. During peak usage periods, the maximum daily water supply can reach up to 1.3 × 10^6^ m^3^. Throughout the experimental period, the annual water supply from Jihongtan Reservoir totaled 3.52 × 10^8^ m^3^.

Two sites in the reservoir were selected for water sampling: site S1 (120°12′28′′ E; 36°20′15′′ N), located at the inlets, and site S2 (120°14′55′′ E; 36°22′22′′ N), located at the outlet. Sampling was conducted from each site monthly for one year, with 10 L water samples collected at 0.5 m below the water surface. After sampling, water temperature (WT), pH, and dissolved oxygen (DO) were determined in situ using a multi-parameter water quality sonde (HQ40d, HACH, Loveland, CO, USA), and the water samples were then transported to the laboratory at 4 °C within 2 h.

Each water sample was divided into two portions, one of which was utilized for measuring water quality parameters, and the other was subjected to DNA extraction. Specifically, physicochemical parameters, including chemical oxygen demand (COD), ammonium nitrogen (NH_4_^+^-N), nitrite nitrogen (NO_2_^−^-N), nitrate nitrogen (NO_3_^−^-N), total nitrogen (TN), and total phosphorus (TP), were determined according to the Environmental Quality Standards for Surface Water in China (GB3838–2002) [10]. Chlorophyll-a (Chl-a) was measured by acetone extraction spectrophotometry, according to Li et al. [27]. A portion of the water samples were filtered through 0.22 μm filter membranes using vacuum filtration devices to collect microorganisms, and collected membranes were stored at −80 °C until DNA was extracted.

### 2.2. DNA Extraction, PCR Amplification, and Sequencing

The DNA of the samples was extracted using the E.Z.N.A.^®^ soil DNA Kit (Omega Bio-tek, Norcross, GA, USA) according to the manufacturer’s instructions. The V3-V4 region of the bacterial 16S rRNA gene was amplified by PCR using primers 338F (5′-ACTCCTACGGGAGGCAGCAG-3′) and 806R (5′-GGACTACHVGGGTWTCTAAT-3′) [28]. The amplicon sequencing was conducted on an Illumina MiSeq platform (Illumina, San Diego, CA, USA) using a paired-end (2 × 250 bp) approach according to the standard protocols by Majorbio Bio-Pharm Technology Co. Ltd. (Shanghai, China). Raw FASTQ files were de-multiplexed using an in-house perl script and then quality-filtered and merged by fastp (v 0.19.6) and FLASH (v 1.2.7), respectively. The quality-filtered sequences with a 97% similarity threshold were clustered into operational taxonomic units (OTUs) using UPARSE v7.1 [29], with the most abundant sequence per OTU selected as the representative. The taxonomy of each representative sequence was analyzed against the 16S rRNA database (Silva v138) by the Ribosomal Database Project (RDP) classifier (release 11.5) [30] using a confidence threshold of 0.7.

### 2.3. Statistical Analysis

The Venn diagram was generated using shared and unique OTUs to depict the similarities and differences within the community. A one-way analysis of variance (ANOVA) with a post-hoc Tukey’s Honest Significant Difference (HSD) test was used to analyze the differences in water physicochemical properties and alpha diversity indices across different seasons. Statistical significance was set at *p*  < 0.05. Non-metric multidimensional scaling (NMDS) was performed for the ordination analysis of planktonic bacterial communities. Analysis of similarity (ANOSIM) was employed to investigate the variability in planktonic bacterial communities between groups. A heatmap of the top 50 genera was constructed using the “ComplexHeatmap” package (v 3.20) in R [31]. The Mantel test was performed to assess the correlations between environmental factors and planktonic bacterial communities by using the “linkET” package (v 0.0.7.4) [32]. We implemented distance-based redundancy analysis (db-RDA) to further reveal the primary environmental variables influencing changes in the planktonic bacterial community structure by using the “vegan” package (v 2.6-10).

To investigate the interactions between planktonic bacterial species, the co-occurrence network analysis was performed with a correlation coefficient (r) ≥ 0.8 and statistical significance at *p* < 0.05. The computation of network properties and identification of keystone taxa were performed using the R package “ggClusterNet” (v 0.1.0) [33]. The network was visualized using Gephi (v 0.9.2). According to Zi (within-module connectivity) and Pi (among-module connectivity) values, nodes in a network can be categorized into four groups: network hubs (Zi > 2.5, Pi > 0.62) are nodes that are highly connected both within their own module and to other modules; module hubs (Zi > 2.5, Pi < 0.62) are nodes that are strongly connected only within a single module; connectors (Zi < 2.5, Pi >0.62) are nodes that connect different modules; and peripherals (Zi < 2.5, Pi < 0.62) are nodes that have limited connectivity to other nodes. Among these, network hubs, module hubs, and connectors have been recognized as potential keystone species [34].

The neutral community model [35] and null model [36] were used in R to investigate the relative impact of stochastic and deterministic processes on planktonic bacterial community assembly. The neutral community model was performed to analyze the contribution of neutral processes in planktonic bacterial community assembly by using the “stats4” (v 3.6.2) and “hmisc” (v 5.2-2) packages [37]. To quantitatively assess the contributions of various ecological processes to the structure of planktonic bacterial communities, the null model based on the beta nearest taxon index (βNTI) and the Bray–Curtis-based Raup–Crick index (RCbray) was calculated using the R packages ‘picante’ and ‘vegan’, respectively [13]. The ecological processes were categorized into deterministic processes and stochastic processes. Deterministic processes include homogeneous selection (βNTI < −2) and heterogeneous selection (βNTI > 2), and stochastic processes include dispersal limitation (|βNTI| < 2 and RCbray > 0.95), homogenizing dispersal (|βNTI| < 2 and RCbray < −0.95), and undominated processes (|βNTI| < 2 and |RCbray| < 0.95) [38].

## 3. Results

### 3.1. Water Physicochemical Parameters

As abiotic conditions, environmental factors can significantly influence bacterial community dynamics and are crucial factors influencing community assembly [38]. To investigate the correlation between environmental factors and seasonal changes in planktonic bacterial communities, the seasonal variations in water physiochemical parameters were analyzed (Figure S1). Except for NH_4_^+^-N, physiochemical parameters showed significant differences among the seasons (*p* < 0.05). The maximum average value of WT occurred in summer (26.03 °C), while the minimum average value was observed in winter (5.17 °C), but no significant differences were found between spring and winter or between summer and autumn. On the contrary, average DO values significantly differed between summer and winter, with the highest average in winter (11.55 mg/L) and the lowest one in summer (5.97 mg/L). The maximum values for both pH and COD occurred in summer (8.51 and 12.8 mg/L, respectively), while the minimum values were observed in spring for pH (8.11) and in autumn for COD (9.8 mg/L). Extreme average values of some environmental factors occurred in autumn. Specifically, mean values of NO_3_^−^-N and TN were lowest in autumn (0.64 mg/L and 1.03 mg/L, respectively), while NO_2_^−^-N, TP, and Chl-a were highest in autumn (0.026 mg/L, 0.033 mg/L, and 19.00 mg/m^3^ respectively).

### 3.2. Composition and Seasonal Succession of Planktonic Bacterial Communities

**Figure 1:**
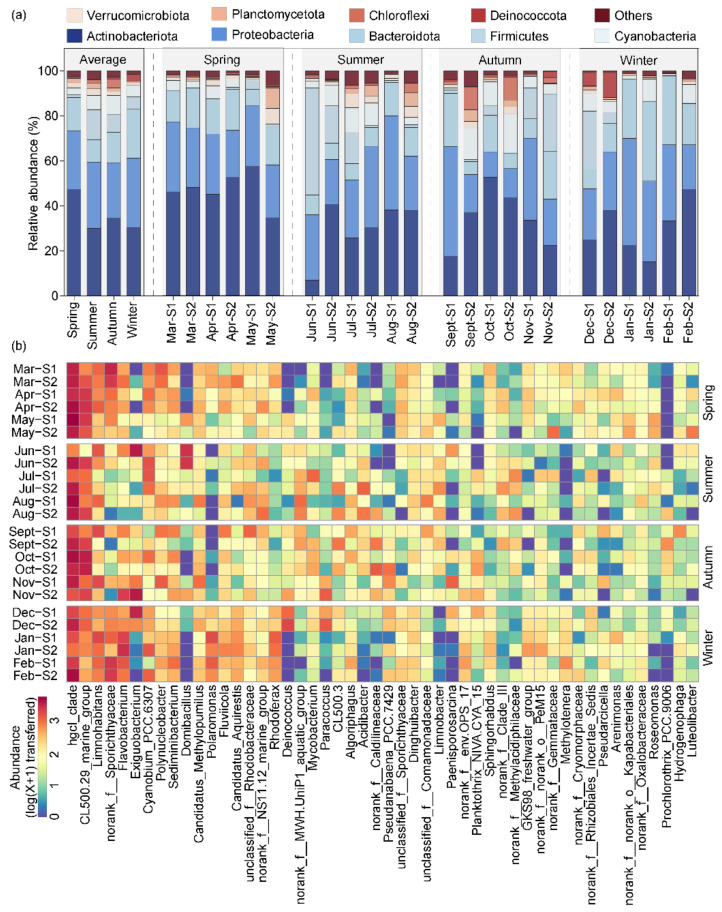
Compositions of the planktonic bacterial community on phyla level (**a**) and genus level (**b**) in Jihongtan Reservoir. Relative abundance less than 1% is defined as others.

In all the water samples, a total of 39 bacterial phyla were identified, and the planktonic bacterial community composition of the top 1% phyla is shown in Figure 1a. The dominant taxa at the phylum level were Actinobacteriota (35.53%), Proteobacteria (27.70%), Bacteroidota (15.10%), Firmicutes (6.85%), Cyanobacteria (5.84%), Verrucomicrobiota (2.00%), Planctomycetota (1.66%), Chloroflexi (1.53%), and Deinococcota (1.05%). The relative abundance of planktonic bacterial dominant phyla showed seasonal dynamics. Actinobacteriota was the predominant bacterial phylum in spring (47.38%), summer (29.98%), and autumn (34.51%); in winter, it accounted for 30.24%, making it the second most dominant phylum. Proteobacteria exhibited a relative abundance of 30.92% in winter, slightly surpassing Actinobacteriota to become the most dominant phylum. However, in spring (25.99%), summer (29.45%), and autumn (24.45%), it ranked as the second most dominant bacterial phylum. Bacteroidota was the third most dominant phylum in spring (14.76%), autumn (13.75%), and winter (21.97%), and its dominance declined in the summer, when it became the fourth most dominant phylum with a relative abundance of 9.90%. Firmicutes was the third dominant phylum in summer (13.48%), and its dominance gradually decreased with seasonal changes, resulting in abundances of 7.66% in autumn, 5.18% in winter, and dropping to 1.10% in spring. Cyanobacteria had the highest relative abundance in autumn (8.66%) and the lowest relative abundance in spring (3.02%). The average relative abundance of Chloroflexi increased gradually from winter to autumn, with respective values of 0.17%, 0.47%, 1.38%, and 4.10%. Verrucomicrobiota and Planctomycetota were dominant in spring, summer, and autumn, but not in winter, when they accounted for only 0.93% and 0.74%, respectively. However, Deinococcota displayed an opposite trend as it was dominant only during winter.

A total of 907 bacterial genera were identified throughout all the water samples, and the planktonic bacterial community composition of the top 50 genera is shown in Figure 1b. The dominant taxa at the gene level were *hgcI_clade* (18.65%), *CL500-29_marine_group* (8.63%), *Limnohabitans* (4.24%), *Flavobacterium* (3.70%), *Exiguobacterium* (3.30%), *Cyanobium_PCC-6307* (3.14%), *Polynucleobacter* (2.02%), *Sediminibacterium* (1.73%), *Domibacillus* (1.57%), *Candidatus_Methylopumilus* (1.43%), *Polaromonas* (1.38%), *Fluviicola* (1.28%), *Candidatus_Aquirestis* (1.23%), *Rhodoferax* (1.18%), and *Deinococcus* (1.04%). Across four seasons, *hgcI_clade*, belonging to Actinobacteriota, was the dominant genus, with a relative abundance of 23.75% in spring, 18.19% in summer, 17.84% in autumn, and 14.81% in winter. *CL500-29_marine_group,* belonging to Actinobacteriota, was the second most dominant genus in spring (11.05%), summer (7.04%), and autumn (12.54%), with a significant decrease in dominance in winter (3.89%). *Flavobacterium,* belonging to Bacteroidota, was the second most dominant genus in winter (7.97%), and its dominance declined in spring (2.20%) and autumn (3.79%) and was lowest in summer (0.83%). *Limnohabitans* belonging to Proteobacteria was the third most dominant genus in spring (4.05%) and winter (5.44%), whereas the third dominant genus in summer and autumn belonged to Firmicutes, specifically *Domibacillus* (5.28%) and *Exiguobacterium* (5.12%), respectively. *Cyanobium_PCC-6307* was the only dominant genus of Cyanobacteria, with relative abundances of 2.88%, 4.69%, 1.34%, and 3.65% from spring to winter, respectively. In addition, *Polaromonas* (4.61%), belonging to Proteobacteria, and *Deinococcus* (2.83%), belonging to Deinococcota, were dominant only in winter, while *Sediminibacterium* (0.86%) and *Fluvicola* (0.59%), both belonging to Bacteroidota, lost their predominance only in the summer.

### 3.3. Alpha Diversity and Beta Diversity of Planktonic Bacterial Communities

**Figure 2:**
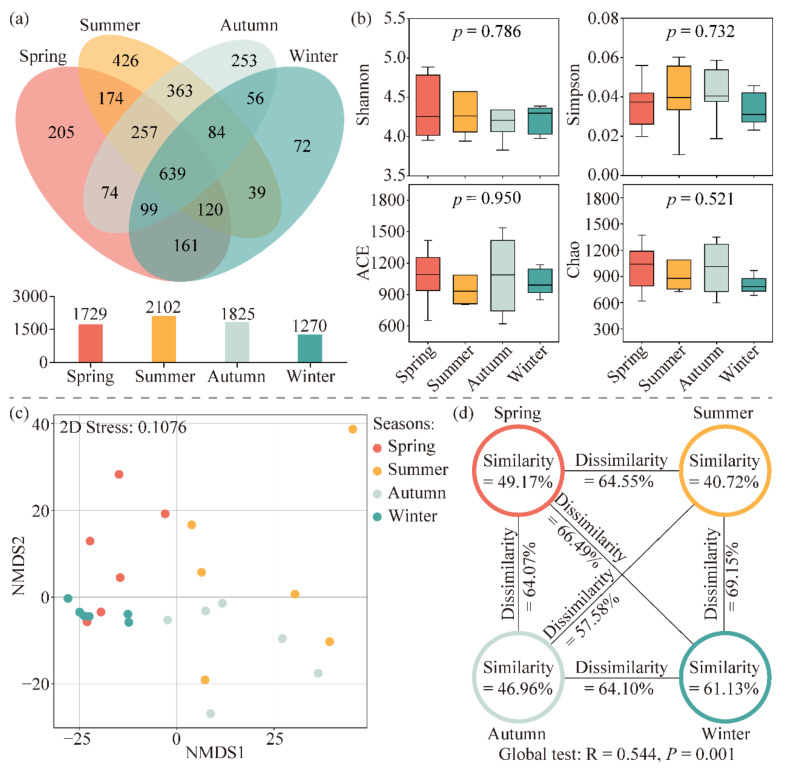
OTUs, alpha diversity, and beta diversity in Jihongtan Reservoir. (**a**) Venn diagram of the OTUs among the four seasons and (**b**) alpha diversity indices of planktonic bacterial communities in the four seasons. (**c**) Non-metric multidimensional scaling analysis (NMDS) ordination plot produced based on Aitchison distance and (**d**) analysis of similarity (ANOSIM) tests.

The high-throughput sequencing produced a total of 753,024 high-quality valid sequences from all samples, and 3022 OTUs were obtained by merging the high-quality sequences with 97% similarity. The Venn diagram showed the similarity and overlap of OTUs in the planktonic bacterial communities across the four seasons (Figure 2a). From spring to winter, 1729, 2102, 1825, and 1270 OTUs were identified, of which 639 OTUs were shared among the four seasons, with 37.0%, 30.4%, 35.0%, and 50.3% of the four groups, respectively. The unique OTUs for the four seasons were 205, 426, 253, and 72, which accounted for 11.9%, 20.2%, 13.9%, and 5.7% in the respective seasons.

The alpha diversity of planktonic bacterial communities was represented using Shannon, Simpson, ACE, and Chao indices. According to Figure 2b, an analysis of the alpha diversity indices across the four seasons indicated variations from season to season, but no significant differences were observed (*p* > 0.05). Specifically, the mean values of the ACE and Simpson indices were highest in autumn, while the Shannon and Chao indices exhibited their maximum mean values in summer and spring, respectively. The mean values of the four alpha diversity indices were at their lowest in winter.

NMDS ordination and ANOSIM tests were conducted to exhibit the beta diversity of planktonic bacterial communities in different seasons (Figure 2c,d). The results of the NMDS analysis showed that the planktonic bacterial communities in the four seasons were distinctly segregated (Stress = 0.1076). The ANOSIM test further illustrated the similarities and differences among the various subgroups, revealing that the planktonic bacterial communities exhibited significant variations across the four seasons (Global R = 0.544, *p* = 0.001). Specifically, the similarity of planktonic bacterial communities within the four seasons was measured at 49.17% in spring, 40.72% in summer, 46.96% in autumn, and 61.13% in winter. The communities exhibited distinct differences between all four seasons, with the most significant variation occurring between summer and winter at 69.15%, while the smallest difference was observed between summer and autumn at 57.58%.

### 3.4. Environmental Factors Influencing Planktonic Bacterial Community Structure

**Figure 3:**
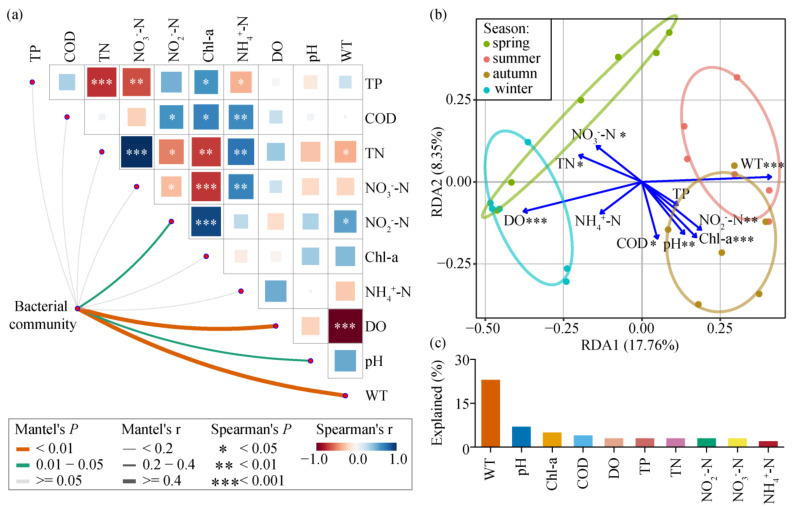
Environmental factors affecting planktonic bacterial communities in Jihongtan Reservoir. (**a**) Mantel tests were used to evaluate the correlations between the planktonic bacterial community and each environmental factor. Pairwise comparisons of environmental factors are visually represented using a color gradient to indicate Spearman’s correlation coefficients. (**b**) Distance-based redundancy analysis (db-RDA) revealed the environmental factors driving planktonic bacterial communities across four seasons. Blue arrows represent different environmental factors. Significance level: *p* < 0.001 ***; *p* < 0.01 **; and *p* < 0.05 *. (**c**) The explanatory value of environmental factors to community.

The Mantel test (Figure 3a) revealed that planktonic bacterial community composition was significantly correlated with WT (r = 0.441, *p* = 0.001), pH (r = 0.441, *p* = 0.001), DO (r = 0.441, *p* = 0.001), and NO_2_^−^-N (r = 0.441, *p* = 0.001) throughout the year (Table S2). The db-RDA results (Figure 3b) demonstrated that the seasonal variation of the planktonic bacterial community structure was related to eight environmental factors, namely, WT, DO, Chl-a, pH, NO_2_^−^-N, TN, NO_3_^−^-N, and COD, and the first two axes explained 8.35% and 17.76% of the variability, respectively. According to Figure 3c, WT was the primary factor that accounted for the seasonal succession of the planktonic bacterial community in Jihongtan Reservoir, explaining 23% of the variation in the planktonic bacterial community structure. The VPA results revealed that environmental factors accounted for 31% of the variation in the planktonic bacterial communities.

### 3.5. Co-Occurrence Networks of Planktonic Bacterial Communities

**Figure 4:**
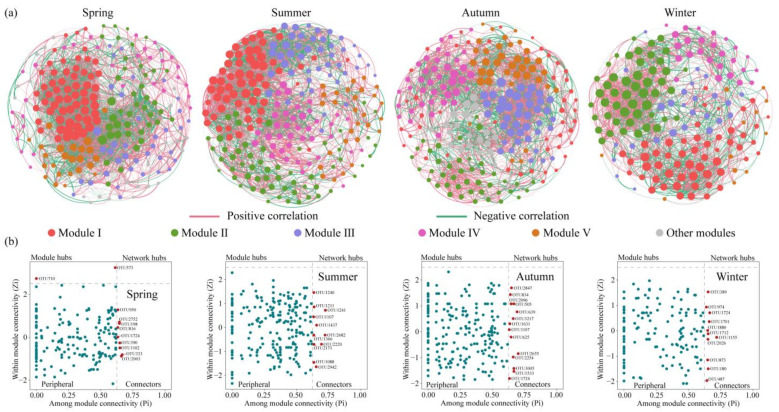
Seasonal co-occurrence network patterns of planktonic bacterial communities in Jihongtan Reservoir. (**a**) Co-occurrence networks across four seasons and (**b**) keystone species analysis across four seasons.

In addition to environmental factors, biological interactions are essential in shaping bacterial community assembly. Figure 4a revealed the co-occurrence network patterns of planktonic bacterial communities based on the OTU level across four seasons, and the topological parameters are listed in Table S3. The four co-occurrence networks, from spring to winter, comprised 216 nodes with 3319 edges, 228 nodes with 2828 edges, 225 nodes with 2745 edges, and 166 nodes with 1666 edges, respectively. The average degrees of the co-occurrence networks were 30.73 in spring, 24.81 in summer, 24.40 in autumn, and 20.07 in winter. Across the four networks, the spring network exhibited the greatest complexity, while the winter network displayed the least complexity. Additionally, the highest graph density, shortest path length, and highest clustering coefficient were observed in the spring network, indicating that the planktonic bacterial community in spring had a more compact network, producing more connections and interactions, with greater efficiency in the transfer of information, energy, and matter within the community. Compared to summer and autumn networks, the winter network had a higher graph density, shorter path length, and lower clustering coefficient, indicating that the planktonic bacterial community in winter was not sufficiently well-connected locally but had higher global connectivity and efficient information transfer. The modularity values of planktonic bacterial networks were 0.35 in spring, 0.49 in summer, 0.47 in autumn, and 0.46 in winter, indicating that the spring network had the lowest modular structure. The four co-occurrence networks displayed predominantly positive correlations among planktonic bacterial microorganisms, with the strongest positive correlation observed in autumn (60.44%) and the weakest one in spring (53.12%), with correlations of 57.60% in summer and 58.34% in winter.

Based on the Zi (within-module connectivity) and Pi (among-module connectivity) values, we identified two module hubs and 9 connectors in spring, 11 connectors in summer, 14 connectors in autumn, and 11 connectors in winter (Figure 4b), all of which could be regarded as keystone nodes. Table S4 provides more detailed taxonomic information about keystone nodes, showing that the key species varied among the four seasonal communities.

### 3.6. Assembly Process of Planktonic Bacterial Communities Across Four Seasons

**Figure 5:**
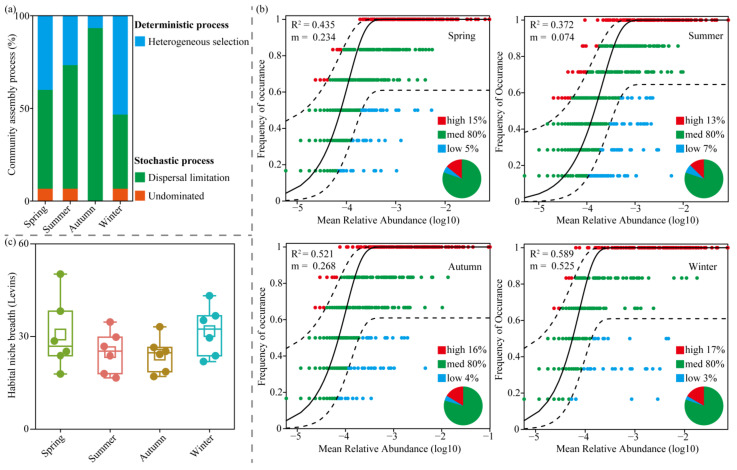
Assembly of planktonic bacterial communities in Jihongtan Reservoir. (**a**) Relative contributions of various ecological processes in assembling the planktonic bacterial community across four seasons. (**b**) Fit of the neutral community model across four seasons; m represents the estimated migration rate, and R^2^ represents the fit to the neutral community model. The solid black line represents the best fit of the neutral community model, while the dashed black lines indicate the 95% confidence interval. (**c**) The niche breadths of planktonic bacterial communities across four seasons.

To explore the assembly mechanism of planktonic bacteria communities during the water diversion period in Jihongtan Reservoir, the null model and neutral model analysis were used (Figure 5a,b). Null model analysis calculated the relative contributions of deterministic and stochastic processes in shaping the planktonic bacterial community, showing that stochastic processes were dominant in spring, summer, and autumn, while deterministic processes have a more crucial role in shaping the winter planktonic bacterial community. We found that heterogeneous selection and dispersal limitation were the main processes affecting planktonic bacteria community assembly during the water diversion period. From spring to winter, heterogeneous selection represented 40.00%, 26.66%, 6.67%, and 53.33%, respectively, while dispersal limitation accounted for 53.33%, 66.67%, 93.33%, and 40.00% respectively. According to the results of the neutral model, the migration rate (m) was higher in winter than in other seasons, reflecting the planktonic bacteria in winter had higher dispersal ability. Furthermore, the average values of niche breadth (Levins) in spring and winter were 30.63 and 31.73, respectively, which were higher than in summer (24.94) and autumn (24.12) (Figure 5c).

## 4. Discussion

### 4.1. Seasonal Dynamics of Planktonic Bacterial Diversity and Community Structure

Planktonic bacterial communities exhibited distinct seasonal successions. During the summer, the proportion of shared OTUs with other seasons was the lowest, while the proportion of unique OTUs was the highest, with the opposite trend in winter. Previous studies have indicated that with an increase in temperature, the metabolic activity and population growth rate of microorganisms exhibit exponential growth, leading to a higher species turnover at higher temperatures [39]. Bacteria that fluctuate with seasonal changes possess unique metabolic functions and resource preferences, which can regulate the variations in biogeochemical processes and ecosystem functioning over time [40]. For instance, *Flavobacterium* was not dominant in the summer but was enriched in the winter due to its cold-tolerant characteristics, which enable it to secrete cryoprotective extracellular polymeric substances in low-temperature environments [41]. In addition, the planktonic bacterial community assembly mechanisms could further explain the microbial dissimilarity [42]. When planktonic bacterial communities are driven by heterogeneous selection, species with varying fitness are filtered through distinct selective forces, resulting in increased beta diversity [43]. Moreover, dispersal limitation would result in aggregated distributions of species in space, leading to more dissimilar structures among communities [42,44].

Previous research on microorganisms in lakes, rivers, and reservoirs has demonstrated that seasonal variations significantly influence microbial diversity [45,46,47]. It is generally accepted that warmer environments are more conducive to microbial growth, enhancing microbial diversity [32]. However, we found that the diversity of planktonic bacterial communities did not change significantly with the seasons during the water diversion period. Continuous water transfers may reduce the impact of seasonal variations on microbial diversity. Some studies have indicated that water transfer projects can change local biodiversity in the water-receiving area, with the majority of these studies observing an increase in biodiversity [48,49,50]. Lv et al. [51] reported that both water diversion and seasonal variations impact planktonic bacterial diversity, with water diversion having a more pronounced effect than seasonal variations. Liu et al. [18] suggested that water transfer connected naturally isolated water bodies, facilitating bacterial dispersal between different habitats and thereby enhancing the diversity of bacterial communities in the water-receiving area. During the water diversion period, the planktonic bacteria in Jihongtan Reservoir can maintain stable species diversity and richness across different seasons, which is beneficial for maintaining the stability and function of the ecosystem [52,53].

### 4.2. Effects of Environmental Factors on Planktonic Bacterial Communities

In this study, the Mantel test and RDA ordination were performed to analyze the correlation between environmental variables and the planktonic bacterial community structure. During the water diversion period, WT, pH, DO, and NO_2_^−^-N were observed to be significantly associated with the planktonic bacterial microbial community structure. Except for NH_4_^+^-N, all other environmental factors exhibited significant seasonal variations, indicating notable environmental heterogeneity of the water. Among these factors, WT, DO, Chl-a, pH, NO_2_^−^-N, TN, NO_3_^−^-N, and COD were found to be correlated with the seasonal changes in planktonic bacterial communities. WT had the highest explanation for changes in planktonic bacterial community structure, making it the most crucial factor in accounting for the variation in the planktonic bacterial community at the seasonal scale. Additionally, ANOSIM tests conducted between planktonic bacterial communities across the four seasons revealed that the greatest dissimilarity occurred between summer and winter, while the least dissimilarity was found between summer and autumn. Correspondingly, the environmental factor WT displayed a comparable pattern of variation, with the highest average values in summer and the lowest ones in winter, showing no significant differences between summer and autumn. This consistent seasonal variation in both planktonic bacterial communities and WT further suggests that temperature is the primary environmental driver of planktonic bacterial seasonal succession in Jihongtan Reservoir. Previous studies have identified temperature as a key factor influencing bacterial community composition [37], which may be due to the fact that temperature, through its influence on microbial enzyme activity and functional metabolism during seasonal changes, promotes the succession of bacteria communities [54]. In addition, pH and DO played an important role in structuring the reservoir communities. pH may affect bacterial growth, metabolism, and other essential activities by impacting enzyme synthesis [55]. DO affects the growth of microorganisms and determines the direction of redox reactions, thereby influencing the abundance of functional genes [56]. Nutrients like TN, TP, NO_3_^−^-N, and NO_2_^−^-N can influence the trophic state of aquatic environments, thereby affecting the structure of microbial communities [57].

### 4.3. Co-Occurrence Network Patterns Across Different Seasons

The findings have revealed that environmental factors in Jihongtan Reservoir vary significantly across different seasons, and this considerable environmental heterogeneity often creates more niche spaces and habitat types, enabling a wider variety of microbial communities to coexist [58]. We discovered that the co-occurrence patterns of the planktonic bacterial communities were variable across seasons, with the spring network being the most complex and the winter network being the simplest. However, networks in both seasons displayed higher connectivity, indicating stronger interactions or niche sharing among microorganisms in spring and winter [25]. Simultaneously, heterogeneous selection processes were found to be significantly more dominant in spring and winter compared to summer and autumn. Our findings support the perspective proposed by Huber et al. [59] that the network associations seem to be more interconnected under heterogeneous selection dominance, suggesting heterogeneous selection plays a significant role in structuring more interconnected network associations.

In the co-occurrence network, higher robustness, higher network complexity, more negatively correlated links, and more keystone taxa could contribute positively to microbial network stability. Research indicates a positive correlation between microbial network complexity and stability [23,26]. Positive correlations among communities create species interdependence, and those that rely on feedback loops may collapse when collectively responding to environmental changes [60]. In contrast, a greater number of negative interactions could help stabilize the asynchronous dynamics [61]. Keystone taxa are vital for the stability of microbial communities, and their removal may result in the collapse of these networks [62]. The robustness results show that the spring network (0.243 ± 0.036), summer network (0.257 ± 0.035), and autumn network (0.267 ± 0.032) demonstrated higher robustness compared to the winter network (0.213 ± 0.047). Furthermore, the highest complexity and strongest negative correlation were observed in the spring network, while the autumn network had the greatest number of key species. During the water diversion period, the amount of water transfer received by the reservoir is constantly changing, with much higher transfers in the spring and winter than in the summer and autumn. However, the stability of the co-occurrence network is higher in spring and lower in winter. This indicates that the stability of the network is not significantly affected by the changes in water diversion. It is likely that, being under long-term water diversion, the co-occurrence network has developed adaptability and become less sensitive to changes in water diversion. Our results may be explained by the findings of Hou et al. [13], who studied the impacts of long-term water diversion on the phytoplankton communities and found that the stability of the co-occurrence network shifted from short-term disruption to long-term adaptation. The analysis revealed that seasonal changes more significantly impact co-occurrence network patterns than water diversion. Additionally, under increased water diversion, more complex networks exhibit greater stability.

### 4.4. Planktonic Bacterial Community Assembly Changes Within Four Seasons During Water Diversion

Across the four seasons, although both stochastic and deterministic processes governed planktonic bacterial community assembly, the relative importance of the two processes varied over time. On one hand, water diversion could cause the alteration of hydrological regimes [9], and as the hydrological connectivity changes, the relative importance of the ecological processes may vary over time [59]. On the other hand, the assembly processes changed with the different seasons, which is similar to previous studies [37,63,64]. In our study, stochastic processes dominated planktonic bacterial community assembly in spring, summer, and autumn, while deterministic processes dominated in winter. In summer and autumn, the higher temperatures may impose environmental constraints that promote increased dispersal limitations [19]. Ecologists suggest that stochastic assembly processes (e.g., dispersal) are more influential in benign environments, whereas deterministic processes (e.g., environmental filtering) should be more common in harsh environments [65]. Finally, niche breadth has been recognized as a major determinant of differences in microbial assembly mechanisms [63,66]. Niche breadth refers to the sum of various resources that an organism can utilize and can be used to infer the species tolerance to changing conditions so that the larger the value, the stronger the adaptive capacity of the corresponding species to different environmental conditions [67,68]. It is generally believed that microorganisms with wider niche breadths typically exhibit higher metabolic plasticity, thus being less influenced by environmental selection, and are more controlled by stochastic processes [69,70]. However, our results are not in line with previous findings. In this study, planktonic bacteria observed in spring and winter presented larger niche breadths on average. Nonetheless, we observed that heterogeneous selection was enhanced and dispersal limitation was reduced during these two seasons. These inconsistent results may be attributed to water diversion. Previous studies have found that niche breadth in winter is narrower than in summer [37,71], while the wider niche breadth observed in spring and winter in Jihongtan Reservoir is likely related to water diversion. We observed that water transfers were significantly higher in spring and winter than in summer and autumn, and increased water diversion enhanced the strength of hydraulic exchange, thereby promoting the migration rate [18]. This has the potential to increase species coexistence by facilitating more species to reach and colonize habitats [72]. On the other hand, increased water diversion brings more nutrients, and some studies have pointed out that the increase in nutrients can reduce interspecific competition, leading to an expansion of community niche width [22]. Additionally, with the increase in water diversion during spring and winter, the role of dispersal limitation in planktonic bacterial community assembly is weakened. Fan et al. [73] demonstrated that microorganisms with a wider niche breadth tend to exhibit higher passive dispersal probabilities and are less influenced by dispersal limitation. In summary, the results indicate that both seasonal changes and water diversion significantly influence the dynamics of planktonic bacterial community assembly.

Previous research suggested that deterministic processes dominate bacterial community assembly in isolated lakes and deep aquifers [74,75], while stochastic processes exhibit greater influence on assembly in interconnected aquatic ecosystems [9,76]. During water diversion, we found that although the deterministic processes and stochastic processes together govern planktonic bacterial community assemblies during the water diversion period, the stochastic processes exhibited a greater influence on planktonic bacterial communities. Specifically, heterogeneous selection and dispersal limitation emerged as the dominant process of planktonic bacterial community assembly. Heterogeneous selection refers to selection under heterogeneous abiotic and biotic environmental conditions, and dispersal limitation refers to the restriction of individual movement to and/or establishment of individuals (colonization) in a new location [38]. Environmental filtering and biotic interactions can also affect the movement and successful establishment of organisms [77]. Furthermore, Wu et al. [78] demonstrated that dispersal limitation can arise through interactions with environmental heterogeneities. Thus, it can be seen that both ecological processes are related to interspecific interactions and environmental heterogeneity in Jihongtan Reservoir. Although water diversion can increase the migration rate of microorganisms, dispersal is limited, likely due to the selection pressures exerted by local biotic and abiotic factors. Studies have found that high immigration rates rarely overwhelm local selection in lakes [79]. During the water diversion period, although stochastic processes dominate in planktonic bacterial community assembly, heterogeneous selection and dispersal limitation are mainly influenced by deterministic processes such as interspecific interactions and environmental filtering. This allows the community to maintain stability and adaptability even in complex and variable environments [10].

## 5. Conclusions

In this study, we used 16S rDNA amplicon sequencing to investigate the seasonal dynamics and assembly of planktonic bacterial communities in Jihongtan Reservoir during year-round water diversion. During water diversion, the seasonal effect on the alpha diversity of planktonic bacteria was not significant, but significant seasonal differences in planktonic bacterial communities were observed. Water temperature was the primary environmental factor driving seasonal succession in planktonic bacterial communities. Moreover, co-occurrence patterns of the planktonic bacterial community showed seasonal variations. In conditions of significantly increased water diversion, more complex co-occurrence networks exhibit higher stability. Stochastic processes dominated community construction in spring, summer, and autumn, while deterministic processes were prominent in winter. Heterogeneous selection and dispersal limitation were the primary drivers of planktonic bacterial community assembly, suggesting environmental filtering and biotic interactions play an important role in the assembly of planktonic bacterial communities in Jihongtan Reservoir. Our study highlights the significant seasonal heterogeneity in environmental factors and its impact on planktonic bacterial community succession, coexistence patterns, and assembly during water diversion. This study expands our understanding of how planktonic bacteria assembly in the storage reservoirs at the end of long-distance water transfer projects across different seasons. Additionally, it provides valuable insights for a comprehensive investigation of the complex ecological effects stemming from water diversion projects. Future studies could incorporate functional metagenomics to elucidate the metabolic capabilities and ecological roles of microbial communities, thereby gaining a more comprehensive understanding of bacterial community dynamics.

## Data Availability

The original contributions presented in this study are included in the article/Supplementary Materials. Further inquiries can be directed to the corresponding author.

## Supplementary Materials

The following supporting information can be downloaded at: https://www.mdpi.com/article/10.3390/microorganisms13020465/s1, Figure S1: Seasonal variation of water physicochemical properties in Jihongtan Reservoir; Table S1: Cumulative inflow amount of the water diversion project to Jihongtan Reservoir (108 m3); Table S2: Mantel tests for the correlation between environmental variables and planktonic bacteria based on Spearman’s rank correlation; Table S3: Topological parameters of planktonic bacterial networks in four seasons; and Table S4: Taxonomic information of keystone taxa within four season networks.
