# Supplementary material for: Deciphering Planktonic Bacterial Community Assembly in the Storage Reservoir of the Long-Distance Water Diversion Project
Source: Microorganisms. 2025 Feb 19;13(2):465. doi: 10.3390/microorganisms13020465 (PMC11858334; doi:10.3390/microorganisms13020465)
Supplement: Supplementary file 1 [file microorganisms-13-00465-s001.zip › microorganisms-3461678-supplementary.pdf]

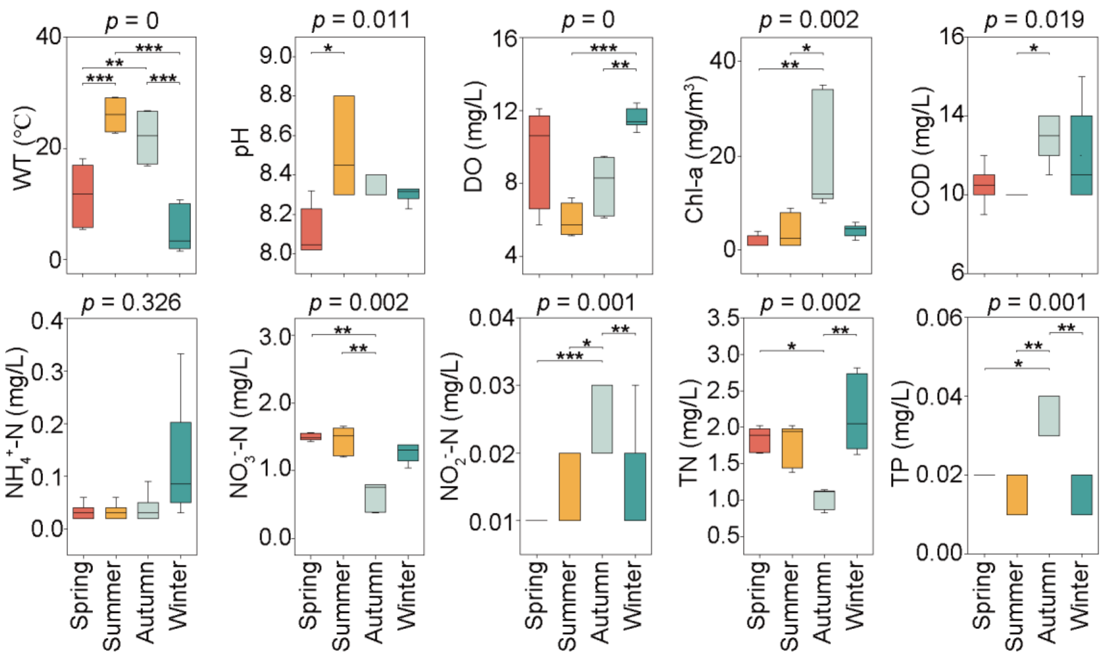

Figure S1. Seasonal variation of water physicochemical properties in Jihongtan Reservoir. (Significance: * $P < 0.05$, ** $P < 0.01$, *** $P < 0.001$)

Table S1. Monthly cumulative inflow amount of water diversion project to Jihongtan Reservoir (10^8 m^3).

Water diversion projects	Spring			Summer			Autumn			Winter		
	**Mar**	**Apr**	**May**	**Jun**	**Jul**	**Aug**	**Sept**	**Oct**	**Nov**	**Dec**	**Jan**	**Feb**
ESNWD	0.221	0.262	0.231	-	-	-	-	0.126	0.034	0.661	0.348	0.205
YQWD	0.064	0.068	0.071	0.255	-	-	-	-	-	-	0.108	0.149
XR	-	-	-	-	0.227	0.057	0.055	0.330	-	-	-	-

Table S2. Mantel tests for the correlation between environmental variables and planktonic bacteria based on Spearman rank correlation.

**Microbes**	**Environmental factors**	**r**	**p**	**Mantel's r**	**Mantel's p**
Planktonic bacteria	WT	0.701	0.001	>= 0.4	< 0.01
	NO_2^--N	0.149	0.045	< 0.2	0.01-0.05
	NO_3^--N	0.030	0.329	< 0.2	>= 0.05
	NH_4^+-N	-0.158	0.987	< 0.2	>= 0.05
	TN	0.076	0.174	< 0.2	>= 0.05
	COD	-0.107	0.954	< 0.2	>= 0.05
	TP	0.098	0.124	< 0.2	>= 0.05
	Chl-a	0.134	0.111	< 0.2	>= 0.05
	DO	0.650	0.001	>= 0.4	< 0.01
	pH	0.200	0.021	< 0.2	0.01-0.05

Table S3. Topological parameters of planktonic bacterial networks in four seasons.

Topological Parameters	Spr	Sum	Aut	Win
Number of Nodes	216	228	225	166
Number of Edges	3319	2828	2745	1666
Average Degree	30.731	24.807	24.400	20.072
Average weighted degree	27.281	21.937	21.587	17.738
Graph Density	0.143	0.109	0.109	0.122
Average Path Length	2.571	2.747	2.761	2.649
Average Clustering Coefficient	0.590	0.563	0.554	0.544
Modularity	0.350	0.490	0.471	0.461
Network diameter	7	7	6	6
Positive correlation (%)	53.12%	57.60%	60.44%	58.34%
Negative correlation (%)	46.88%	42.40%	39.56%	41.66%

Table S4. Taxonomic information of keystone taxa within four seasons networks.

Group	Role	OTUs	relative abundance	Phylum	Genus
Spring	Module hubs	OTU573	2.92%	Bacteroidota	*Flavobacterium*
		OTU710	0.18%	Bdellovibrionota	*Peredibacter*
	Network hubs	NA	NA	NA	NA
	Connectors	OTU198	0.14%	Planctomycetota	*CL500-3*
		OTU333	0.08%	Proteobacteria	*alphaI_cluster*
		OTU390	0.35%	Bacteroidota	*Solitalea*
		OTU816	0.11%	Bacteroidota	*OLB12*
		OTU950	0.90%	Actinobacteriota	*unclassified*
		OTU1102	0.15%	Cyanobacteria	*Cyanobium_PCC-6307*
		OTU1724	0.23%	Proteobacteria	*alphaI_cluster*
		OTU2003	0.14%	Proteobacteria	*Arenimonas*
		OTU2752	0.11%	Cyanobacteria	*Cyanobium_PCC-6307*
Summer	Module hubs	NA	NA	NA	NA
	Network hubs	NA	NA	NA	NA
	Connectors	OTU1088	0.23%	Cyanobacteria	*Cyanobium_PCC-6307*
		OTU1107	1.68%	Proteobacteria	*Acidibacter*
		OTU1211	0.47%	Proteobacteria	*norank_f__MWH-UniP1_aquatic_group*
		OTU1240	0.07%	Bdellovibrionota	*norank*
		OTU1241	0.15%	Actinobacteriota	*norank*
		OTU1366	0.13%	Bacteroidota	*norank*
		OTU1437	0.65%	Actinobacteriota	*Mycobacterium*
		OTU2171	0.46%	Proteobacteria	*unclassified*
		OTU2220	0.05%	Bdellovibrionota	*OM27_clade*
		OTU2682	0.06%	Proteobacteria	*unclassified*
		OTU2942	0.16%	Bacteroidota	*norank_f__Cryomorphaceae*
Autumn	Module hubs	OTU1601	0.34%	Unassigned	*Unassigned*
	Network hubs	NA	NA	NA	NA
	Connectors	OTU505	0.06%	Cyanobacteria	*Cyanobium_PCC-6307*
		OTU625	0.22%	Proteobacteria	*unclassified*
		OTU639	1.03%	Cyanobacteria	*Planktothrix_NIVA-CYA_15*
		OTU834	0.09%	Bacteroidota	*norank_f__NS11-12_marine_group*
		OTU1107	0.39%	Proteobacteria	*Acidibacter*
		OTU1533	1.93%	Proteobacteria	*Candidatus_Methylopumilus*

		OTU1631	0.14%	Chloroflexi	*norank*
		OTU1724	0.15%	Proteobacteria	*alphaI_cluster*
		OTU2096	0.09%	Firmicutes	*Fictibacillus*
		OTU2254	0.05%	Proteobacteria	*unclassified*
		OTU2655	0.38%	Actinobacteriota	*Pseudarthrobacter*
		OTU2847	1.52%	Proteobacteria	*unclassified*
		OTU3005	0.23%	Bacteroidota	*Dinghuibacter*
		OTU3217	0.12%	Bacteroidota	*norank_f__NS11-12_marine_group*
Winter	Module hubs	NA	NA	NA	NA
	Network hubs	NA	NA	NA	NA
	Connectors	OTU180	0.42%	Proteobacteria	*norank_f__Methylophilaceae*
		OTU389	1.35%	Proteobacteria	*Rhodoferax*
		OTU487	0.18%	Bacteroidota	*Dinghuibacter*
		OTU873	0.22%	Planctomycetota	*norank_f__Gemmataceae*
		OTU974	0.12%	Verrucomicrobiota	*Terrimicrobium*
		OTU1155	1.65%	Cyanobacteria	*Cyanobium_PCC-6307*
		OTU1701	0.24%	Proteobacteria	*Acinetobacter*
		OTU1712	0.05%	Proteobacteria	*Hyphomonas*
		OTU1724	0.15%	Proteobacteria	*alphaI_cluster*
		OTU1880	0.12%	Bacteroidota	*Flavobacterium*
		OTU2026	0.08%	Proteobacteria	*norank_f__Comamonadaceae*

NA: Not Available
